# Active acoustic illusions for stealth and subterfuge

**DOI:** 10.1038/s41598-019-49828-0

**Published:** 2019-09-19

**Authors:** Daniel Eggler, Nicole Kessissoglou

**Affiliations:** School of Mechanical and Manufacturing Engineering, The University of New South Wales, Sydney, 2052 Australia

**Keywords:** Mechanical engineering, Acoustics

## Abstract

Acoustic illusion devices present a novel approach for defeating detection systems such as sonar by misrepresenting information about the target. These devices are currently designed for a predetermined illusion using metamaterials. We present the first active acoustic illusion utilizing monopole control sources and error sensors arranged circumferentially around a rigid object to generate the desired illusion in the global acoustic field. We also utilize control sources and error sensors in a line array to generate an illusion in the forward-scatter region of the object. Multiple types of illusions are achieved for a given control configuration.

## Introduction

The last decade has seen an unprecedented level of control for electromagnetic and acoustic wave manipulation. Pendry *et al*.^1^ pioneered the field of transformation optics which resulted in a surge of exciting developments including cloaking^2–5^ and illusions^6–21^. Cloaking renders an object invisible whilst illusions misrepresent the true nature of an object being perceived. Yang *et al*.^6^ proposed the first optical illusion using a metamaterial shell to increase the scattered field of an object, making the object appear larger than its actual size. In contrast, Jiang *et al*.^7^ employed the use of metamaterials to visually shrink an object. Lai *et al*.^8^ created an illusion device using two distinct metamaterial shells, where one shell comprised negative permittivity and negative permeability to cancel an optical region, whilst the other shell comprised a homogenous medium with positive anisotropic permeability to project an illusion within the cancelled region. Using a similar approach, Jiang *et al*.^13^ experimentally developed a radar illusion device comprising two metamaterial shells which was able to modify the scattered field of a metallic cylinder to be that of a dielectric cylinder. McManus *et al*.^18^ proposed an illusion device in which a subwavelength coating of an anisotropic material was applied to a flat plate to reproduce the scattering properties of a curved surface, and vice versa. Recently, Yang *et al*.^21^ proposed a dual purpose homogenous illusion device, which could either cloak an object or misrepresent its location.

Transformation optics was soon generalized to acoustics which allowed for manipulation of the scattered field using analogous counterparts, to achieve acoustic cloaking^22–34^ and acoustic illusions^35–37^. Kan *et al*.^35^ utilized positive anisotropic metamaterials to generate acoustic illusions near boundaries of curved geometry. They numerically and experimentally demonstrated the transformation of scattered waves from a cylinder to appear as scattered waves from a rectangular prism^35^. Liu and He^37^ also employed positive isotropic materials to exchange the acoustic scattering potential of an object with that of the desired illusion.

The aforementioned optical and acoustical illusions were generated employing passive approaches, primarily using metamaterials. As an alternative, active techniques have also been utilized to create optical illusion effects^38–41^. Ma *et al*.^38^ experimentally produced an illusional field for a defined region on a conducting plate, by modulating the voltage of control sources arranged circumferentially around the region. Similar to our active approach, Cao *et al*.^39^ employed different control configurations comprising closed and open arrangements in which control sources completely circumscribed the object or were localised to certain areas. Using a fixed control configuration, they demonstrated the ability to generate different optical illusions that misrepresented either the orientation or location of the object. Similar to the work by Cao *et al*.^39^, we herein employ a fixed control configuration to generate single-type illusions; however we further demonstrate the ability to generate multiple illusions simultaneously. Zheng *et al*.^40^ numerically presented an active control configuration comprising three multipole sources surrounding an apple-shaped object, to create the optical illusion of the scattered field corresponding to a banana-shaped object. Our work extends the capability by Zheng *et al*.^40^ to actively generate an illusion such that the acoustic field arising from scattering by a rigid body for a given incident excitation can be replaced with a different incident field.

Active control techniques for acoustic cloaking have also been implemented, see^42–46^; however, no active approaches have been employed to generate acoustic illusions. Herein we present the first active acoustic illusions utilizing arrays of monopole control sources and error sensors to misrepresent the acoustic fields due to scattering by a rigid object. Acoustic illusions are generated to change the apparent size of the object, shift the location of the object, and to simultaneously change the size and location of the object. Unlike their passively produced counterparts, the aforementioned active acoustic illusions are achieved with no required changes to the control configuration. Further, we actively modify the acoustic response of an object due to an incident field to resemble a different incident field.

## Results

### Active control configuration and theory

**Figure 1:**
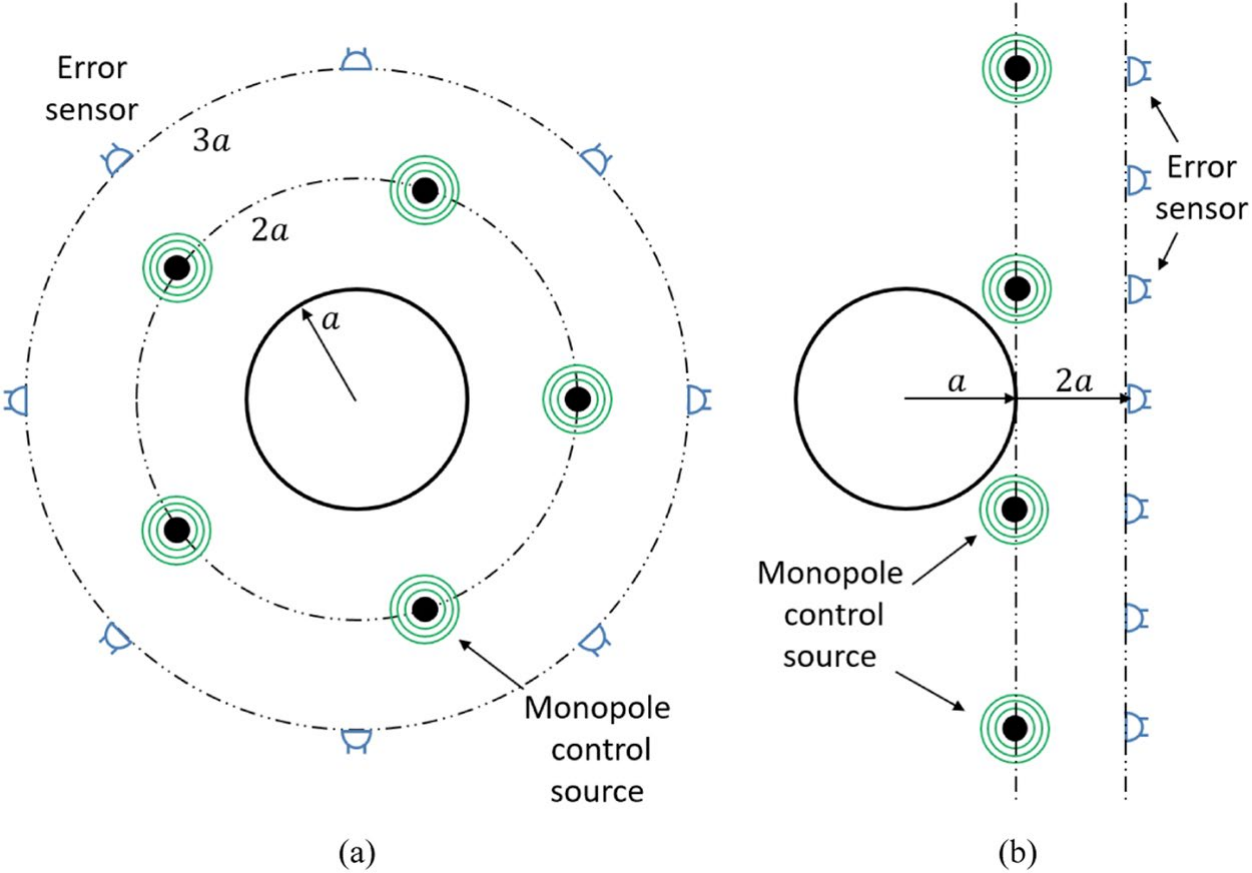
Control configurations comprising monopole control sources and microphone error sensors placed (**a**) circumferentially around a rigid cylinder at 2*a* and 3*a*, respectively, and (**b**) in a line array at *a* and 2*a*, respectively.

Let’s consider a time-harmonic incident excitation corresponding to either a plane wave or a single monopole source impinging on a rigid 2D cylinder of radius *a*. The acoustic field arising from scattering by the incident excitation is herein known as the primary acoustic field. We implement a multichannel control configuration utilising *W* control sources and *L* error sensors. Figure 1 shows the two active control configurations considered in our study, comprising arrays of monopole control sources and microphone error sensors located circumferentially around the object to generate an illusion in the global acoustic domain (Fig. 1(a)), and line arrays of control sources and error sensors adjacent to the object to generate illusions in the forward-scatter direction (Fig. 1(b)).

The Helmholtz equation governs the acoustic domain for time harmonic responses. The acoustic pressure due to scattering by a rigid cylinder arising from either an incident plane wave or monopole source excitation is well established, for example, see^47^. Our active control strategy requires modification to a cost function to accommodate a two-stage process. The first stage is used to completely remove the total acoustic field at the error sensor locations (sound cancellation). The second stage is then employed to generate the desired acoustic illusion (sound reproduction). The cost function for cancellation of the total acoustic field is defined as the sum of the squared acoustic pressures at the error sensor locations, given by

1 $$J={{\bf{e}}}^{{\rm{H}}}{\bf{e}},$$

where the superscript ()^H^ denotes the Hermitian transpose operator and **e** is a vector denoting the total acoustic field at the error sensors arising from scattering by both the incident excitation and the secondary monopole control sources. This vector can be expressed as

2 $${\bf{e}}={{\bf{p}}}_{{\rm{p}}}+{\bf{Zq}},$$

where **p**_p_ denotes the primary acoustic pressure at the *L* error sensor locations, **Z** is an *L* × *W* matrix of complex transfer functions representing the secondary field due to *W* monopole control sources at *L* error sensor locations, and **q** is the vector of *W* complex control source strengths to be optimized upon minimization of the total acoustic pressure given by Eq. (2). For a rigid body, the primary acoustic pressure is given by **p**_p_ = **p**_inc_ + **p**_sc_, where **p**_inc_ is a vector denoting the incident acoustic pressure and **p**_sc_ is a vector denoting the scattered pressure by the rigid body due to **p**_inc_. Substituting Eq. (2) into Eq. (1) and differentiating the resulting expression with respect to the real and imaginary components of the control source strengths, the following optimal control source strengths are obtained as

3 $${\bf{q}}=-\,{[{{\bf{Z}}}^{{\rm{H}}}{\bf{Z}}]}^{-1}{{\bf{Z}}}^{{\rm{H}}}\,{{\bf{p}}}_{{\rm{p}}}.$$

In the second stage, the acoustic illusional field is generated. The vector incorporating the acoustic pressure of the illusion at the error sensor locations becomes

4 $${\bf{e}}={{\bf{p}}}_{{\rm{p}}}+{\bf{Zq}}-{{\bf{p}}}_{{\rm{I}}},$$

where **p**_I_ is a vector representing the acoustic pressure of the desired illusion at the error sensor locations. Following the same procedure described previously, the optimal control source strengths now become

5 $${\bf{q}}=-\,{[{{\bf{Z}}}^{{\rm{H}}}{\bf{Z}}]}^{-1}{{\bf{Z}}}^{{\rm{H}}}({{\bf{p}}}_{{\rm{p}}}-{{\bf{p}}}_{{\rm{I}}}).$$

A further simplification to Eq. (5) can be implemented if the acoustic illusion only requires manipulation of the scattered field. Such a situation arises when the desired acoustic illusion is generated by the secondary acoustic field superimposing with the original incident field. In this case, the first stage is modified to actively cancel the scattered field only, and the optimal control source strengths now become

6 $${\bf{q}}=-\,{[{{\bf{Z}}}^{{\rm{H}}}{\bf{Z}}]}^{-1}{{\bf{Z}}}^{{\rm{H}}}({{\bf{p}}}_{{\rm{sc}}}-{{\bf{p}}}_{{\rm{I}}}).$$

### Illusions that actively manipulate the scattered field

**Figure 2:**
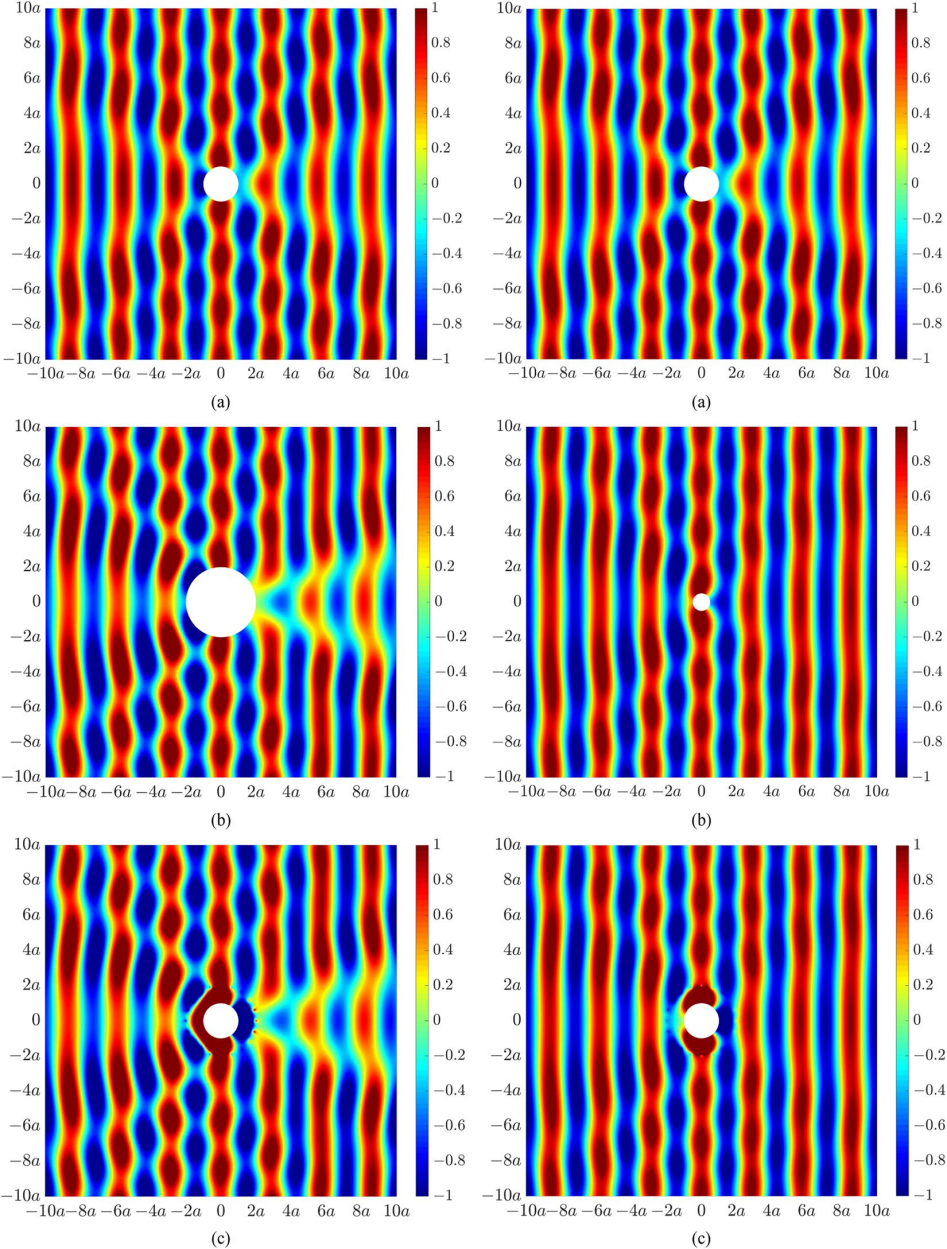
Acoustic pressure fields under plane wave excitation at 200 Hz for (**a**) a rigid cylinder of radius *a*, (**b**) a rigid cylinder of radius 2*a* (left) and 0.5*a* (right), and (**c**) a rigid cylinder of radius *a* with an actively modified acoustic field to resemble the acoustic field for a cylinder of radius 2*a* (left) or 0.5*a* (right).

Two different excitation cases are considered corresponding to a plane wave propagating from left to right, or a single monopole source located 4*a* upstream of the cylinder (in the back-scatter region) and horizontally aligned with the cylinder centroid, both at 200 Hz. We herein show results for acoustic pressure in Pascal. Figure 2(a) presents the primary acoustic pressure field arising from scattering due to an incident plane wave, showing clear signs of scattering upstream and downstream of the cylinder. Figure 2(b) presents the acoustic fields due to scattering of the incident plane wave by a cylinder of radius 2*a* (left) and 0.5*a* (right), showing greater and reduced scattering effects due to the larger and smaller sized object, respectively. Figure 2(c) presents the acoustic illusional fields whereby the primary acoustic field in Fig. 2(a) of the original cylinder of radius *a* has been actively modified to resemble the acoustic fields in Fig. 2(b) for a cylinder of radius 2*a* (left) or 0.5*a* (right). A global acoustic illusion in the exterior acoustic field transpires beyond the control sources located at 2*a*.

**Figure 3:**
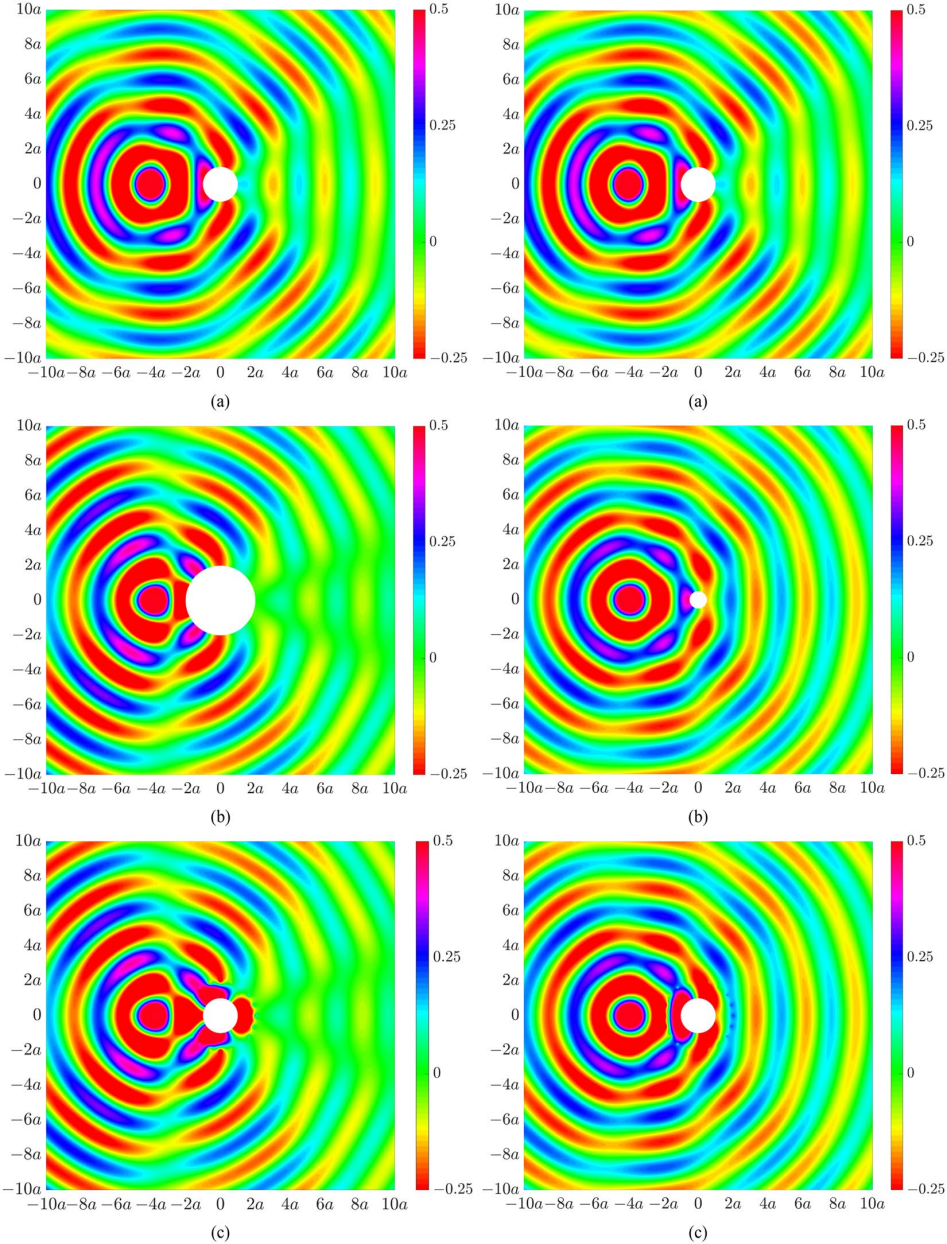
Acoustic pressure fields under monopole source excitation at 200 Hz for (**a**) a rigid cylinder of radius *a*, (**b**) a rigid cylinder of radius 2*a* (left) and 0.5*a* (right), and (**c**) a rigid cylinder of radius *a* with an actively modified acoustic field to resemble the acoustic field for a cylinder of radius 2*a* (left) or 0.5*a* (right).

Figure 3 presents similar results for the second excitation case corresponding to a single primary monopole source located at 4*a* in the back-scatter region. Figure 3(a) shows the primary acoustic pressure field for our original cylinder of radius *a*, in which the effect of cylindrical spreading can be clearly observed. Figure 3(b) presents the desired acoustic fields associated with scattering of the monopole source by a larger sized cylinder of radius 2*a* (left) and a smaller sized cylinder of radius 0.5*a* (right). Figure 3(c) presents the acoustic illusions. Beyond the control source locations, the actively modified field of the original cylinder in Fig. 3(a) now resembles the acoustic response associated with the larger cylinder (Fig. 3(c), left) and smaller cylinder (Fig. 3(c), right).

**Figure 4:**
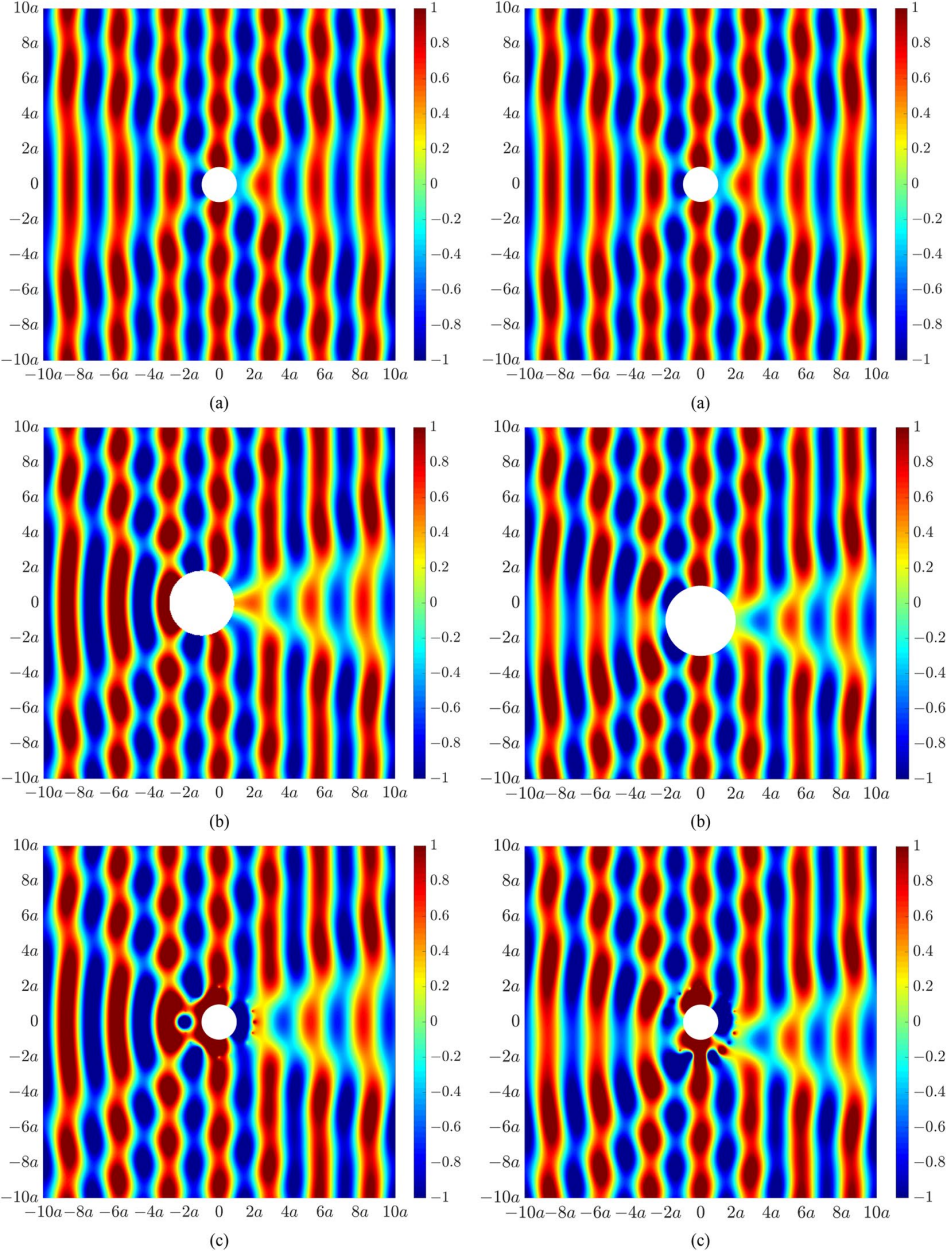
Acoustic pressure fields at 200 Hz for (**a**) a rigid cylinder of radius *a*, (**b**) a rigid cylinder of radius 2*a* shifted to the left by a distance *a* (left) or shifted down by a distance *a* (right), and (**c**) a rigid cylinder of radius *a* with an actively modified acoustic field to resemble the acoustic field for a cylinder of radius 2*a* shifted to the left by a distance *a* (left) or shifted down by a distance *a* (right).

We now generate an active illusion which misrepresents both the size and location of the object. Similar to the results in Fig. 2(a), Fig. 4(a) presents the primary acoustic field arising from an incident plane wave impinging on a rigid cylinder of radius *a*. The targeted acoustic fields now correspond to that of a larger sized cylinder of radius 2*a* shifted to the left (in the back-scatter region) by a distance *a* (Fig. 4(b), left) or shifted normal to the direction of sound propagation by a distance *a* (Fig. 4(b), right). Figure 4(c) presents the actively modified acoustic fields of the original cylinder of radius *a* such that the resultant acoustic fields beyond the control source locations now correspond to the responses associated with a cylinder of different size and location.

**Figure 5:**
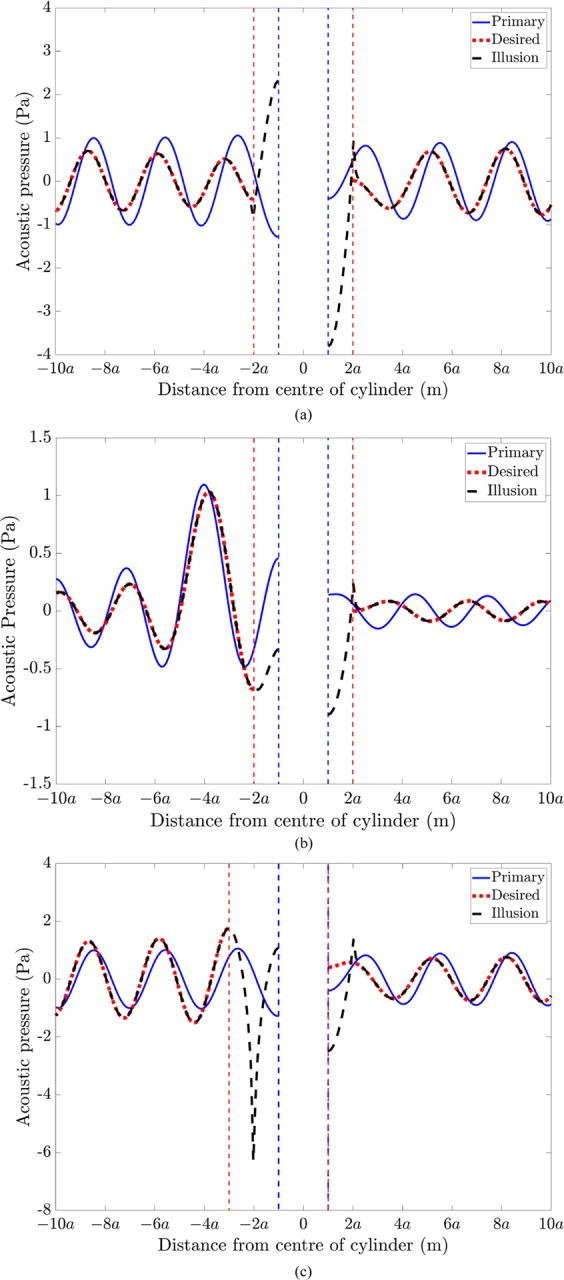
Acoustic pressure as a function of distance from the rigid cylinder along *θ* = 0, showing the primary acoustic pressure (solid blue line), desired acoustic pressure (dotted red line) and the actively generated illusion pressure (dashed black line), for (**a**) a rigid cylinder of radius *a* under plane wave excitation to resemble the acoustic field for a cylinder of radius 2*a* (as per Fig. 2(c), left), (**b**) a rigid cylinder of radius *a* under monopole excitation to resemble the acoustic field for a cylinder of radius 2*a* (as per Fig. 3(c), left), and (**c**) a rigid cylinder of radius *a* under plane wave excitation to resemble the acoustic field for a cylinder of radius 2*a* and shifted to the left by a distance *a* (as per Fig. 4(c), left). The dashed blue and red vertical lines respectively represent the perimeter of the rigid cylinder and the illusion cylinder.

We herein compare the primary acoustic pressure, the desired pressure and the actively generated illusion pressure, as a function of distance from the cylinder centre along *θ* = 0. In Fig. 5, the primary acoustic pressure arises from scattering by a rigid cylinder of radius *a* due to plane wave excitation (Fig. 5a,c)) or due to a single primary monopole source located in the back-scatter region at −4*a* (Fig. 5(b)). As per the acoustic illusion field in Fig. 2(c)(left), the desired and actively generated illusion pressures in Fig. 5(a) correspond to the acoustic pressure for a cylinder of radius 2*a* under plane wave excitation. Similarly, the desired and actively generated illusion pressures in Fig. 5(b) correspond to the acoustic pressure for a cylinder of radius 2*a* under monopole source excitation, as per the illusion in Fig. 3(c)(left). For each excitation case, the illusion pressure becomes identical to that of the desired pressure beyond the control source perimeter at 2*a*. The desired and illusion pressures in Fig. 5(c) correspond to the acoustic field for a cylinder of radius 2*a* and shifted to the left by a distance *a*, as per the acoustic illusion field in Fig. 4(c)(left). In the back-scatter region, the illusion pressure becomes identical to that of the desired pressure beyond the perimeter of the illusion cylinder at a radial distance of −3*a*. However, in the forward-scatter region, the illusion pressure does not match the desired pressure at the perimeter of the illusion cylinder corresponding to *a*, but instead matches the desired pressure at 2*a* corresponding to the control source radial location. We observe that when the perimeter of the illusion object is enclosed within the control source perimeter, the illusion field takes effect beyond the control source perimeter. However, when the perimeter of the illusion object exceeds the control source perimeter, the illusion takes effect from the illusion object perimeter, resulting in a reduction of the size of the acoustic illusion domain. When the desired illusion is based on scattering by the same incident field, a global illusion is obtained in both the forward- and back-scatter regions.

### Illusions that actively manipulate the total field

**Figure 6:**
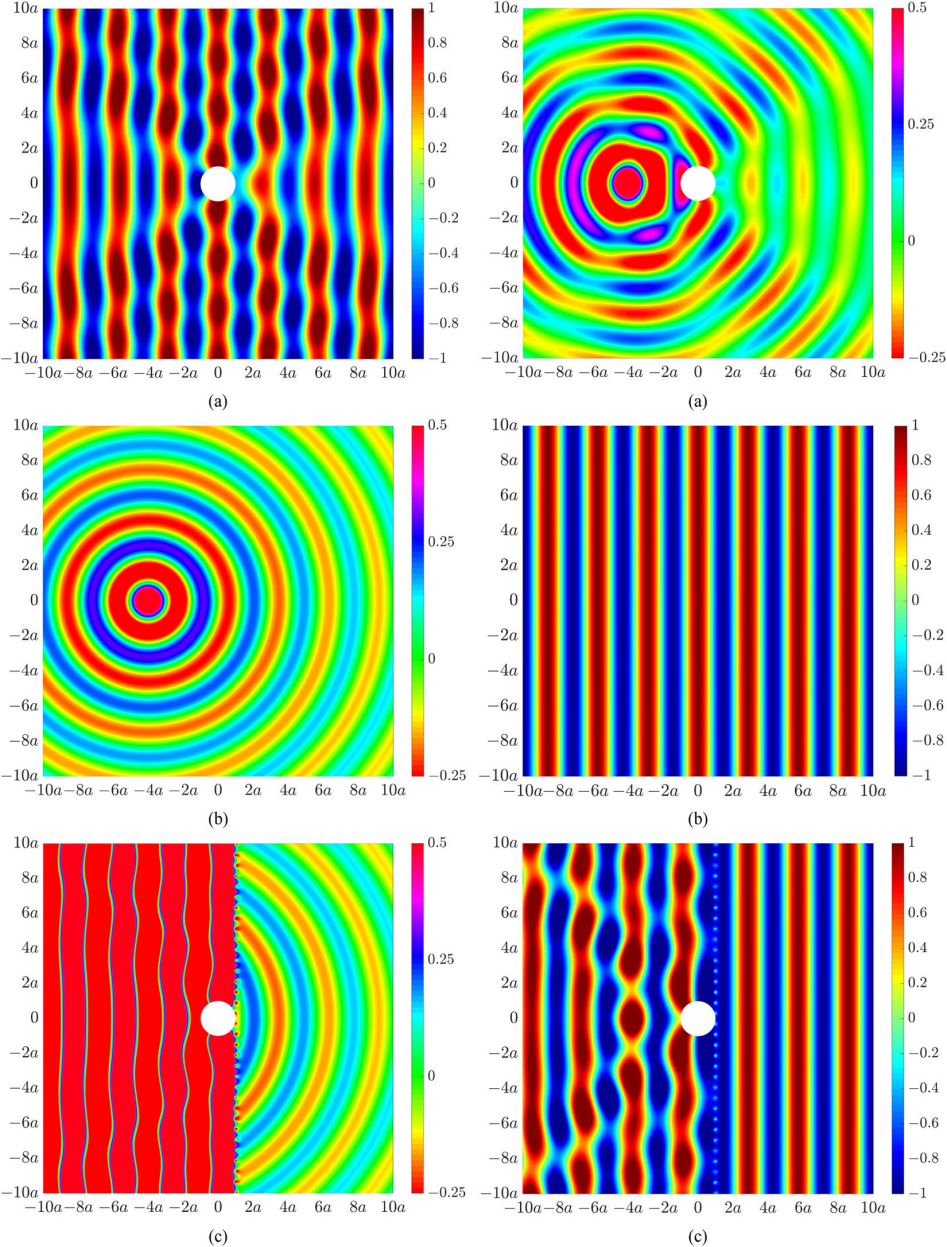
Acoustic pressure fields at 200 Hz for (**a**) a rigid cylinder of radius *a* due to excitation from a plane wave (left) and monopole source (right), (**b**) an incident field for a monopole source (left) and a plane wave (right), (**c**) a rigid cylinder of radius *a* with actively modified acoustic fields to resemble the acoustic field for a monopole source incident field (left) or an incident plane wave (right) in the forward-scatter region.

The acoustic illusions in Figs 2(c)–4(c) were generated using the control configuration shown in Fig. 1(a), utilizing 20 control sources and 40 error sensors arranged circumferentially around the cylinder of radius *a*. As the resultant acoustic fields associated with the targeted illusions were based on the same incident field, the optimal control source strengths were obtained using Eq. (6), whereby only the scattered field of the primary source, **p**_sc_, was actively cancelled. We herein utilize the second control configuration in Fig. 1(b) to generate an acoustic illusion such that the resultant field in the forward-scatter region corresponds to the incident field from a different excitation. Figure 6(a) shows the primary acoustic field arising from scattering by the cylinder of radius *a* due to an incident plane wave (left) and monopole source located 4*a* upstream of the cylinder (right). Figure 6(b) shows the acoustic illusion to be produced in the forward-scatter region of the cylinder, corresponding to an incident field (in the absence of an object) for a monopole source (left) or a plane wave (right). Figure 6(c) presents the actively modified acoustic fields in Fig. 6(a) to produce the desired illusional field effective from the control source line array in the forward-scatter region. As these results require cancellation of the total primary acoustic field (incident and scattered), the optimal control source strengths were obtained using Eq. (5). The size of the domain of the illusional field is strongly dependent on the number and placement of the control sources and error sensors. To generate an effective illusion within the domain shown here, the line arrays were significantly extended, utilizing 200 control sources and 400 error sensors equally distributed along their respectively axes from −60*a* to 60*a*. Further, it was not possible to actively generate an illusional field in the back-scatter region using the line array configuration due to the requirement to cancel the primary incident field.

**Figure 7:**
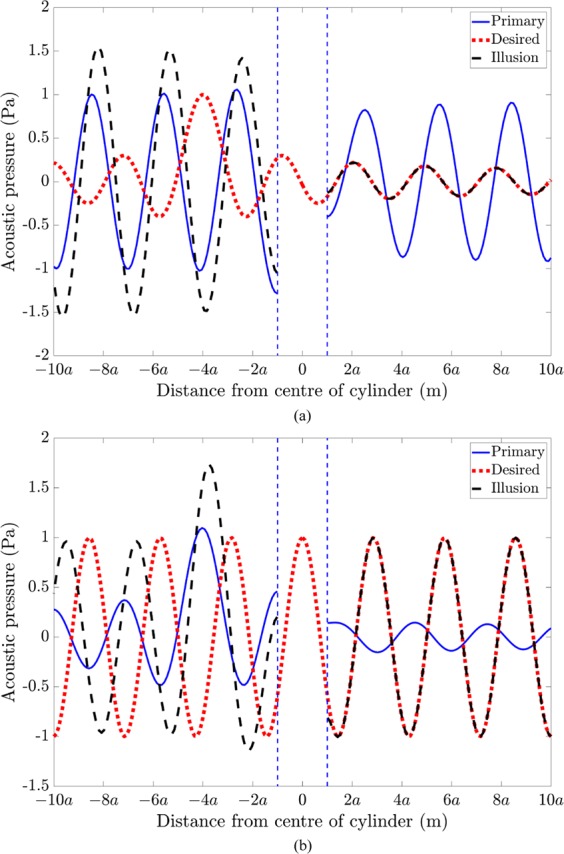
Acoustic pressure as a function of distance from the rigid cylinder along *θ* = 0, showing the primary acoustic pressure (solid blue line), desired acoustic pressure (dotted red line) and the actively generated illusion pressure (dashed black line), for (**a**) a rigid cylinder of radius *a* under plane wave excitation to resemble the incident acoustic field due to a monopole source located at (4*a*, *π*) in the forward-scatter region (as per Fig. 6(c), left), and (**b**) a rigid cylinder of radius *a* under monopole excitation to resemble the incident acoustic field due to a plane wave in the forward-scatter region (as per Fig. 6(c), right). The dashed vertical blue line represents the rigid cylinder perimeter.

Figure 7 compares the primary acoustic pressure, desired pressure and actively generated illusion pressure, as a function of distance from the cylinder centre along *θ* = 0, for the illusions presented in Fig. 6. The primary acoustic pressure arises from scattering by a rigid cylinder of radius *a* due to plane wave excitation (Fig. 7(a)) or a single monopole source located at −4*a* in the back-scatter region (Fig. 7(b)). As per the acoustic illusion field in Fig. 6(b)(left), the desired pressure in Fig. 7(a) corresponds to the acoustic pressure radiated by a monopole source located at −4*a* in the back-scatter region. Similarly, as per the acoustic illusion field in Fig. 6(b)(right), the desired pressure in Fig. 7(b) corresponds to a plane wave. For both cases, the desired pressure assumes that the rigid cylinder does not exist. The illusion pressure in Fig. 7(a,b) is shown to take effect in the forward-scatter region beyond the control source line array located at *a*.

## Discussion

In summary, we present a method to generate active acoustic illusions for a rigid body. An illusion in the global acoustic domain beyond the control source perimeter can be achieved using control sources and error sensors fully circumscribing the object. An illusion in the forward-scatter region was demonstrated using a line array of control sources and error sensors, for which the size of the acoustic domain of the illusion is strongly dependent on the number of control sources and sensors. Using a fixed control configuration, active acoustic illusions were generated for single and multiple type illusions. We also demonstrated the ability to replace the acoustic field arising from scattering by a rigid body for a given incident excitation with a different incident field. The proposed technique depends on prior knowledge of the incident field as well as the primary acoustic field arising from scattering of an incident field by a rigid body. As such, the technique can work effectively for a rigid body of arbitrary shape whereby a numerical solution to obtain the primary and secondary acoustic responses can be implemented. Applications of the proposed technique include masking of unwanted sound and misrepresenting information about a target for stealth purposes.

## Methods

The analytical simulations of the active illusions presented in this paper were conducted within MATLAB. The incident and scattered acoustic fields of the rigid cylinder were approximated using truncated multipole expansions given in^45^. Analytical results for the incident and scattered acoustic fields for both plane wave and monopole source excitation were numerically validated using COMSOL Multiphysics 5.4. The fluid medium was considered to be inviscid and free of losses for the analytical and numerical simulations. The system parameters comprise a 2D rigid cylinder of radius *a* = 0.6 m in air of density *ρ*_*f*_ = 1.225 kg/m^3^ and speed of sound *c*_*f*_ = 343 m/s, with negligible material damping.

## Data Availability

The datasets generated during and/or analysed during the current study are available from the corresponding author on reasonable request.

## References

[1] Pendry JB, Schurig D, Smith DR (2006). Controlling electromagnetic fields. Science.

[2] Cai W, Chettiar UK, Kildishev AV, Shalaev VM (2007). Optical cloaking with metamaterials. Nat. Photon..

[3] Alù A, Engheta N (2008). Plasmonic and metamaterial cloaking: physical mechanisms and potentials. J. Opt. A Pure Appl. Opt..

[4] Farhat M, Guenneau S, Movchan AB, Enoch S (2008). Achieving invisibility over a finite range of frequencies. Opt. Express.

[5] Alù A (2009). Mantle cloak: Invisibility induced by a surface. Phys. Rev. B.

[6] Yang T, Chen H, Luo X, Ma H (2008). Superscatterer: Enhancement of scattering with complementary media. Opt. Express.

[7] Jiang WX, Cui TJ, Yang XM, Ma HF, Cheng Q (2011). Shrinking an arbitrary object as one desires using metamaterials. Appl. Phys. Lett..

[8] Lai Y (2009). Illusion optics: The optical transformation of an object into another object. Phys. Rev. Lett..

[9] Ng J, Chen H, Chan CT (2009). Metamaterial frequency-selective superabsorber. Opt. Lett..

[10] Chen H, Chan CT, Sheng P (2010). Transformation optics and metamaterials. Nat. Mater..

[11] Jiang WX, Ma HF, Cheng Q, Cui TJ (2010). Illusion media: Generating virtual objects using realizable metamaterials. Appl. Phys. Lett..

[12] Li C (2010). Experimental realization of a circuit-based broadband illusion-optics analogue. Phys. Rev. Lett..

[13] Jiang WX, Cui TJ (2011). Radar illusion via metamaterials. Phys. Rev. E.

[14] Xu Y, Du S, Gao L, Chen H (2011). Overlapped illusion optics: a perfect lens brings a brighter feature. New J. Phys..

[15] Liu M, Lei Mei Z, Ma X, Cui TJ (2012). Dc illusion and its experimental verification. Appl. Phys. Lett..

[16] Jiang WX, Qiu CW, Han T, Zhang S, Cui TJ (2013). Creation of ghost illusions using wave dynamics in metamaterials. Adv. Funct. Mater..

[17] Han T, Bai X, Thong JT, Li B, Qiu CW (2014). Full control and manipulation of heat signatures: Cloaking, camouflage and thermal metamaterials. Adv. Mater..

[18] McManus T, Valiente-Kroon J, Horsley S, Hao Y (2014). Illusions and cloaks for surface waves. Sci. Rep..

[19] Yi J, Tichit P-H, Burokur SN, de Lustrac A (2015). Illusion optics: Optically transforming the nature and the location of electromagnetic emissions. J. Appl. Phys..

[20] Guo Y, Yan L, Pan W, Shao L (2016). Scattering engineering in continuously shaped metasurface: An approach for electromagnetic illusion. Sci. Rep..

[21] Yang C, Huang M, Yang J, Mao F, Li T (2018). Target illusion by shifting a distance. Opt. Express.

[22] Chen H, Chan CT (2007). Acoustic cloaking in three dimensions using acoustic metamaterials. Appl. Phys. Lett..

[23] Cummer SA, Schurig D (2007). One path to acoustic cloaking. New J. Phys..

[24] Torrent D, Sánchez-Dehesa J (2007). Acoustic metamaterials for new two-dimensional sonic devices. New J. Phys..

[25] Cheng Y, Yang F, Xu JY, Liu XJ (2008). A multilayer structured acoustic cloak with homogeneous isotropic materials. Appl. Phys. Lett..

[26] Cummer SA (2008). Scattering theory derivation of a 3D acoustic cloaking shell. Phys. Rev. Lett..

[27] Farhat M, Enoch S, Guenneau S, Movchan AB (2008). Broadband cylindrical acoustic cloak for linear surface waves in a fluid. Phys. Rev. Lett..

[28] Norris AN (2008). Acoustic cloaking theory. Proc. R. Soc. A.

[29] Torrent D, Sánchez-Dehesa J (2008). Acoustic cloaking in two dimensions: a feasible approach. New J. Phys..

[30] Cheng Y, Liu XJ (2009). Three dimensional multilayered acoustic cloak with homogeneous isotropic materials. Appl. Phys. A.

[31] Popa B-I, Cummer SA (2009). Cloaking with optimized homogeneous anisotropic layers. Phys. Rev. A.

[32] García-Chocano VM (2011). Acoustic cloak for airborne sound by inverse design. Appl. Phys. Lett..

[33] Popa B-I, Zigoneanu L, Cummer SA (2011). Experimental acoustic ground cloak in air. Phys. Rev. Lett..

[34] Zigoneanu L, Popa B-I, Cummer SA (2014). Three-dimensional broadband omnidirectional acoustic ground cloak. Nat. Mater..

[35] Kan W (2013). Acoustic illusion near boundaries of arbitrary curved geometry. Sci. Rep..

[36] Kan W (2016). Three-dimensional broadband acoustic illusion cloak for sound-hard boundaries of curved geometry. Sci. Rep..

[37] Liu Y, He S (2018). Acoustic illusion using materials with isotropic and positive parameters. Phys. Rev. Appl..

[38] Ma Q, Mei ZL, Zhu SK, Jin TY, Cui TJ (2013). Experiments on active cloaking and illusion for Laplace equation. Phys. Rev. Lett..

[39] Cao, B., Sun, L. & Mei, Z. Realization of radar illusion using active devices. *Adv. Optoelectron* **2012** (2012).

[40] Zheng H, Xiao J, Lai Y, Chan C (2010). Exterior optical cloaking and illusions by using active sources: A boundary element perspective. Phys. Rev. B.

[41] Du J, Liu S, Lin Z (2012). Broadband optical cloak and illusion created by the low order active sources. Opt. Express.

[42] Guevara Vasquez F, Milton GW, Onofrei D (2009). Active exterior cloaking for the 2D Laplace and Helmholtz equations. Phys. Rev. Lett..

[43] Guevara Vasquez F, Milton GW, Onofrei D (2009). Broadband exterior cloaking. Opt. Express.

[44] Cheer J (2016). Active control of scattered acoustic fields: Cancellation, reproduction and cloaking. J. Acoust. Soc. Am..

[45] Eggler D, Chung H, Montiel F, Pan J, Kessissoglou N (2019). Active noise cloaking of 2D cylindrical shells. Wave Motion.

[46] Rajabi M, Mojahed A (2018). Active acoustic cloaking spherical shells. Acta Acust. united Ac..

[47] Martin, P. A. *Multiple scattering: Interaction of time-harmonic waves with N obstacles*. (Cambridge University Press, 2006).

